# The mitochondrial genomes of three species of poison frogs (Anura: *Dendrobates*)

**DOI:** 10.1080/23802359.2017.1347830

**Published:** 2017-07-11

**Authors:** Mariana L. Lyra, Eugenia Sanchez, Sven Künzel, Stefan Lötters, Célio F.B. Haddad, Miguel Vences

**Affiliations:** a Department of Zoology and Aquiculture Centre (CAUNESP), Campus Rio Claro, São Paulo State University – UNESP, Biosciences Institute, Rio Claro, SP, Brazil;; b Zoological Institute, Technical University of Braunschweig, Braunschweig, Germany;; c Department of Evolutionary Genetics, Max Planck Institute for Evolutionary Biology, Plön, Germany;; d Biogeography Department, Trier University, Trier, Germany

**Keywords:** Amphibia, Anura, Dendrobatidae, mitogenomes, RNAseq

## Abstract

We reconstructed nearly complete mitogenomes for three species of poison frogs, *Dendrobates auratus, D. leucomelas*, and *D. tinctorius,* from RNAseq data. We recovered the 13 protein-coding genes, 22 tRNA genes (except tRNA-Val for *D. leucomelas*), and two rRNA genes for all three species, plus partial sequences of the control region. The order of genes agrees with that known from a previously sequenced *D. auratus*, being the most commonly found for neobatrachian frogs. Based on full-sibling comparisons we estimate the probable error rate of Illumina-RNAseq reconstructed mitogenomes of up to 0.15%.

Poison frogs (Dendrobatidae) are a fascinating group of anurans that serve as model to understand the evolution of aposematism and alkaloid sequestering (e.g. Santos et al. 2016). Although their phylogeny has been intensively studied (e.g. Grant et al. 2006; Santos et al. 2009), typically it has been reconstructed based on a limited number of molecular markers. Here we report nearly complete sequences of mitochondrial genomes of three species of *Dendrobates*, reconstructed from transcriptome (RNAseq) data, with the aim to provide additional molecular data that can be used, for example, in future examination of the evolutionary diversification of Poison frogs.

Captive-bred specimens of the species *Dendrobates auratus, D. leucomelas*, and *D. tinctorius* were obtained through the courtesy of Mark Pepper (Understory Enterprises, Charing Cross, Ontario, Canada) and Olivier Dominikowski (DendroWorld, Bullecourt, France). In addition, a wild-caught specimen of *D. leucomelas* was available from Gurí, Bolívar State, Venezuela (8°18’51’’N, 62°39’56’’W); collection permits were issued by Oficina Nacional de Diversidad Biológica (Oficios No. 0908 and No. 0217). Specimens were euthanized using MS-222 overdoses and samples of a mix of different organs (muscle, liver, skin, heart, and lungs) were taken from the freshly sacrificed specimens. They were immediately stored in RNAlater and preserved at −80 °C. Approximately 100 mg of tissue of each sample was used for RNA extraction using a TRIzol protocol. We prepared RNA for sequencing following the Illumina TruSeq stranded mRNA Library Prep protocol. Sequencing was carried out on the Illumina NextSeq (2 × 75 bp paired-end) platform using NextSeq^®^ 500/550 High Output Kit v2.

To assemble mitogenomes from the quality-trimmed Illumina reads, we first normalized raw data to an average depth of 100× using BBNorm (BBMap/BBTools; http://sourceforge.net/projects/bbmap/). The assembly was carried out with MIRA v4.0 (Chevreux et al. 1999) and MITObim v1.8 (Hahn et al. 2013), following Machado et al. (2016) and using the reference mitogenome of *Bufo gargarizans* (NC020048) as initial seed. Assemblies were manually verified in Geneious software v.6 (Biomatters, San Francisco, CA) to evaluate the coverage and quality of each mitochondrial element. Positions with coverage lower than 4 or poly-A regions were coded as ambiguous (‘N’). Preliminary annotation of each sequence was done using the mitochondrial genome annotation server MITOS (Bernt et al. 2013), and the annotation was manually validated by comparison with the available *Dendrobates auratus* mitochondrial genome (JX564862). New sequences were submitted to GenBank (accession numbers MF069434–MF069441). Sequences were aligned to published complete or near-complete mitochondrial genomes of dendrobatid and aromobatid frogs (Zhang et al. 2013; Machado et al. 2016; Vacher et al. 2016) with MAFFT v.7 (Katoh and Standley 2013). The mitogenome sequence of *Bufo tibetanus* was used as outgroup. Maximum-likelihood phylogenetic inference was performed in RAxML v 7.2.7 (Stamatakis 2006) using the model GTRGAMMA and rapid bootstrap heuristics with 100 pseudoreplicates, as implemented in the CIPRES Science Gateway (http://www.phylo.org).

We assembled mitogenomes for one individual of *Dendrobates auratus*, two of *D. leucomelas*, and five of *D. tinctorius,* recovering between 54,083 and 452,667 reads per specimen corresponding to mitochondrial genes. *D. auratus* and *D. tinctorius* had the typical composition of the animal mitochondrial genome, consisting of 13 protein-coding, two ribosomal RNA (rRNA), 22 transfer RNA (tRNA) genes, and the control region in between cytochrome B and tRNA-Leu (D-loop; not completely sequenced). For *D. leucomelas*, we also found the same gene composition, but we were unable to recover tRNA-Val, probably because the region between 12S and 16S had very low coverage. The arrangement of genes was identical for that previously reported for other dendrobatid and aromobatid frogs. For *D. auratus*, we were able to sequence the tRNA cluster LTPF that was not sequenced in the previously published mitochondrial sequence. Most coding genes were encoded on the heavy strand, except for ND6 and eight tRNA genes. The light-strand putative replication origins (O_L_) were found between tRNA-Asn and tRNA-Cys, forming a typical ‘WANO_L_CY’ cluster. Most protein-coding genes used the start codon ATG, but some also used GTG, ATA, ATT, and ATC. The stop codons for four genes (ND1, COII, COIII, and ND3) were T– and should be completed by the addition of 3′ A residues to the mRNA; for other genes, the stop codons were TAA, TAG, AGA, or AGG. The GC content of the mitogenomes, excluding control region, was 42.5% for *D. auratus*, 44% for *D. leucomelas*, and 42.9% for *D. tinctorius*.

We observed 129 nucleotide differences among the mitogenome sequences of a captive *D. leucomelas* and one collected in the wild, and 53 differences between our *D. auratus* sequence and a previously published one (Zhang et al. 2013), corresponding to uncorrected p-distances of 0.9 and 0.4%, respectively. All *D. tinctorius* in our study came from the same captive-bred strain, and specimens Dt3, Dt4, Dt5, Dt6, and Dt9 were full siblings. Their mitochondrial genomes therefore could be expected to be identical. Yet, after removing two short ambiguous regions with poly-A stretches, the full sibling sequences showed 7–22 nucleotide differences among each other, that is, up to 0.15% p-distance. We verified the majority of these differences in the original reads and it is highly unlikely that these differences represent *de-novo* mutations. These values thus probably provide an estimate of the error rate to be expected when assembling mitochondrial genomes of amphibians from RNAseq data sequenced with the chemistry and instruments used herein.

The genus *Dendrobates* contains five species (Grant et al. 2006), three of which are studied in this paper. The phylogenetic tree in Figure 1 recovers the three species in one clade, placing *D. leucomelas* closer to *D. auratus*, thus confirming previous studies (Pyron and Wiens 2011). The complete mitogenomes assembled here provide important DNA data for further phylogenetic and evolutionary analysis of this genus, including our understanding of aposematism and alkaloid sequestering.

**Figure 1.**
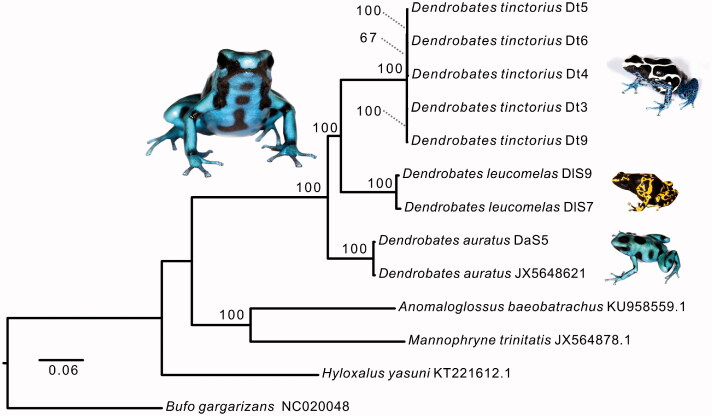
Maximum-likelihood tree constructed under the GTR model from mitogenomic sequences of dendrobatid and aromobatid frogs. Bootstrap support is shown at nodes and numbers following terminal names are unique sample identifiers or GenBank accession numbers.
